# Associations between Perceived Child-Parent Relationships and School Engagement among 9–11 Aged Children

**DOI:** 10.3390/children8070595

**Published:** 2021-07-14

**Authors:** Pirita Markkula, Anja Rantanen, Anna-Maija Koivisto, Katja Joronen

**Affiliations:** 1 Unit of Health Sciences, Tampere University, Kalevantie 4, 33014 Tampere, Finland; anja.rantanen@tuni.fi (A.R.); anna-maija.koivisto@tuni.fi (A.-M.K.); 2 The School of Well-Being and Health Technology, Tampere University of Applied Sciences, Kuntokatu 3, 33520 Tampere, Finland; 3 Department of Nursing Science, University of Turku, 20014 Turku, Finland; katja.joronen@utu.fi

**Keywords:** parent-child relations, school engagement, children, students

## Abstract

School engagement has been shown to protect students from dropping out of education, depression and school burnout. The aim of this Finnish study was to explore the association between child-parent relationships and how much 99,686 children aged 9–11 years liked school. The data were based on the 2019 School Health Promotion Study, conducted by the Finnish Institute for Health and Welfare. This asked children whether they liked school or not and about their child-parent relationships. Univariate and multivariate analyses were used to examine the data separately for boys and girls and the results are presented as odds ratios (OR) and 95% confidence intervals (CI). According to the results, girls showed more school engagement than boys (81.9% versus 74.0%), and it was more common in children who felt that their parents communicated with them in a supportive way. This association was slightly stronger for girls than boys (OR 2.46 95% CI 2.33–2.59 versus OR 2.10 95% CI 2.02–2.20). It is important that child-parent relationships and communication are considered during school health examinations, so that children who have lower support at home can be identified.

## 1. Introduction

School engagement is a multidimensional concept [1,2,3], which has been mainly explored by three aspects, namely emotional, behavioral and cognitive school engagement [2]. Emotional engagement includes a child’s negative and positive feelings towards school and can be measured by asking how much a child likes or dislikes school [2,4]. Behavioral engagement includes grades and other observable actions or performances and cognitive engagement includes a child’s perceptions and beliefs in relation to themselves, their school and their peers [1]. These three types of school engagement are not isolated processes and are dynamically interrelated [2]. Several factors related to sociodemographic factors, teachers, parents and peers are related to school engagement [1,2,3]. These include gender [5,6,7], age [8], the parents’ socioeconomic status and ethnical background [9], teacher support [10], teacher-child relationships [11], parenting style [12], parental involvement [13], parent support [5,6,7,14,15,16,17,18,19], peer support and peer group quality [10].

High levels of school engagement have been shown to predict better school achievements [20,21,22] and higher satisfaction with life [23]. School engagement has also been identified as a protective factor for dropping out of education, depression [20] and school burnout [9,14]. Dropping out of education has been reported to be a risk factor for social exclusion [24]. Child-parent relationships significantly influence a child’s mental health development and their future life [25,26]. Excellent relationships in the family are found to be associated with high mental wellbeing among school-aged children [27]. A number of previous studies have indicated that supportive child-parent relationships had a positive impact on the child’s school engagement [28,29,30,31]. Children need support and guidance from their parents when they start primary school and this persists into puberty [25].

This study measured emotional school engagement by whether children liked school. The same one-item-indicator was used by the World Health Organization (WHO) Health Behaviour in School-aged Children (HBSC) study of 50 countries, which examined children’s engagement with school [32]. According to data on 11-year-old children in the 2020 HBSC study, 43% of girls and 35% of boys liked school a lot in all countries. However, the figures for Finnish children were much lower, with only 21% of girls and 14% of boys reporting they liked school a lot [33]. The Programme for International Students Assessment (PISA) conducted by the Organisation for Economic Co-operation and Development (OECD) investigated school engagement with a one-item-indicator of “sense of belonging at school”, which is related to liking school. According to the 2018 OECD study, 75% of Finnish children reported that they felt that they belonged at school and this was higher than the average rate for the OECD countries, which was 71% [34].

According to previous research, girls initially engage better at school than boys [5,6,7,20], but this declines in both genders as they move to upper grades [7,20,28]. One study found that children who lived with both parents had higher rates of school engagement than those living with one parent [35]. In addition, children from single-parent families engaged better than children who did not live with either of their parents [36]. Several previous studies have showed that having an immigrant background was associated with lower levels of school engagement [15,20,21,37,38]. However, there have been some conflicting results. Some studies have reported that first generation immigrant children had higher levels of school engagement than those whose families had immigrated earlier and native-born children. This phenomenon is called the ”immigrant paradox” [21,39].

Associations between parental social support and school engagement have been widely reported in primary schools [15], junior high schools and high schools, for both genders [5,6,7,14,16,17,18,19]. The social support that parents provide can be measured by asking how often children discuss their lives and school-related matters with their parents [7]. Good quality child-parent relationships have been associated with higher levels of school engagement by boys and girls in primary school, junior high school and high school [28,29,30,31]. High parental monitoring has been shown to have a positive impact on school engagement, which can be measured by asking questions such as whether a parent knows who their child spends their free time with [7,40,41,42,43,44]. Positive associations between parental monitoring and school engagement have been reported for both genders [45] and in single and two-parent families [46]. Previous studies have also shown a positive association between family boundaries and school engagement. Family boundaries can be measured by asking questions such as whether the family provides the child with clear rules [16]. Overall, future research is needed to determine the complex associations between child-parent relationships, school engagement and socio-demographics.

This research focused on child-parent relationships, from the communication perspective. This has rarely been studied in conjunction with school engagement and we were unable to find any Finnish studies that examined this research question. The aim of this study was to use large-scale nationally representative data from nearly 100,000 Finnish children aged 9–11 years to explore the association between the quality of child-parent relationships and school engagement. The research questions were as follows: (1) How the background factors are associated with the school engagement; (2) How the quality of child-parent relationships are associated with the school engagement, and (3) Which factors explain the school engagement?

## 2. Materials and Methods

### 2.1. Data Collection and Measures

Mandatory education starts in Finland in the year that the child turns 7 years old and comprises six years in primary school and three years in junior high school. It is normally preceded by one year of pre-primary education. Children can then go on to upper secondary school, were they can choose between general and vocational education, and then universities and universities of applied sciences [47]. The participants in this study were primary school students.

Nationally representative data were obtained from the 2019 Finnish School Health Promotion (later SHPS) study and comprised 99,686 children (49.9% boys), who were aged 9–11 years and in the 4th and 5th grades. This represented a response rate of 82.0% [48]. The study collects data every other year on the well-being, health and schoolwork of Finnish children and adolescents and the data are used to plan and evaluate school health promotion activities at school at municipal and national levels [49,50]. The data are gathered by anonymous and voluntary questionnaires in classrooms [50] and respondents are supervised by a teacher so that they cannot see each other’s answers [51]. The data were gathered from 4th and 5th graders from comprehensive school and their guardians, 8th and 9th graders from comprehensive school, 1st and 2nd graders from upper secondary school and 1st and 2nd graders from vocational school in year 2019 [50]. In Finland, children in the 4th and 5th grades are approximately 9–11 years old [52].

The current study followed the widely recognized principles of integrity, meticulousness, and accuracy in conducting the research, and presenting, and evaluating, the results.

The data acquisition, research and evaluation methods were ethically sustainable and suitable for the research criteria [53] and complied with Finnish ethical principles on research with human participants [54]. The School Heath Promotion Study was evaluated by the Ethics Committee of Finnish Institute of Health and Welfare in 2018 and met the ethical principles for conducting research [55].

School engagement was examined by asking the respondents one question: “What do you think about school at the moment? I like school”. Respondents were evaluating the item on 4-point scales: “very much”, “quite liked”, “little” and “never”.

Child-parent relationship was assessed by a 4-item scale: “How often this following things happens to you (1) You discuss your school day with your parents (2) You agree the time to come home with your parents when you go out, (3) Your parents talk with your friends when they meet them, (4) Your parents support and encourage you”. Each item was evaluated on 3-point scales: “often”, “sometimes” and “never” [56,57]. A number of background factors were examined, namely gender, school grade, living arrangements and immigrant status [57].

### 2.2. Data Analysis

The original variable of school engagement was described using numbers and percentages. It was also categorized into two groups: “a high level of school engagement” comprised children who liked school: “very much” and “quite liked” and “low level of school engagement” comprised children who liked school “little” and “never”.

Girls were much more likely to have a high level of school engagement than boys according to previous studies [5,7,9]. As a result, we carried out separate gender analyses.

The background factors we used were school grade, living arrangements and immigrant background. The original variable of living arrangements was categorized into four groups: “both parents at home” included “with both parents in the same home”, “shared residency” included “I live same amount of time with both parents, for example every other week, my parents do not live together” and “I live mostly with other parent and sometimes with other parent for example in weekends”, “one parent” included “with one parent” and “neither parent” included “with grandparents or other relatives without my parents”, “foster home”, “children’s home” “youth detention home or approved school”, “residential family home” and “other way”.

The child-parent communication indicator was formed based on the 4-item child-parent relationship scale, which was originally developed by the multidisciplinary specialist group at the Finnish Institute for Health and Welfare. This indicated how supportive and communicative the relationship was between the child and their parent. The child-parent communication indicator was formed from children who responded to all the items in the child-parent relationship scale. The response option to all four were “often” (score 2), “sometimes” (score 1) and “never” (score 0). The scores were then added together to create the child-parent relationship sum variable with a value range from 0 to 8. The Cronbach’s alpha of the child-parent relationship sum variable was 0.59. The sum variable was then categorized into a dichotomous variable (indicator), based on the ratings by multidisciplinary specialist group at the Finnish Institute for Health and Welfare. This indicator is regularly used when reporting the results of SHPS [56]. A value of 6 or more indicated that the child received supportive communication from their parents, and values below that indicated a lower level of supportive communication.

The background factors, namely the child-parent relationship and school engagement, were described by numbers and percentages and the associations between variables were examined using cross-tabulations, the chi-square test and logistic regression. Because of the large sample size (*n* = 99,686), *p*-values of less than 0.01 were considered statistically significant [58,59].

Logistic regression analyses were conducted to evaluate the associations between school engagement and child-parent relationships and the background variables. The dichotomous variable of school engagement was used as a dependent variable and the child-parent communication indicator and background factors were used as explanatory variables. The unadjusted models comprised just one variable each and the adjusted model comprised all the variables, which were simultaneously entered into the model. The results of the logistic regression are presented as odds ratios (ORs) with 95% confidence intervals (95% CIs) and *p*-values. The data were analyzed using IBM SPSS Statistics, version 26 (IBM Corp., New York, NY, USA) [60].

## 3. Results

The background factors of the 4th and 5th grade children, aged 9–11 years, are presented in Table 1 and this shows that the majority (73.2%) were living with both parents.

The children’s engagement with school is presented in Table 2. A high level of school engagement was more prevalent in girls than in boys (*p* < 0.001) and more girls (18.0%) than boys (13.8%) liked school very much.

The associations between the background factors and school engagement are presented in Table 3. Levels of school engagement were higher in the younger 4th grade girls and boys, aged 9–10, than in the 5th grade students, aged 10–11 (*p* < 0.001). School engagement was statistically significantly associated with living arrangements (*p* < 0.001), the school engagement was higher among girls and boys living with both parents than children living with just one parent. It was also higher in immigrant girls and boys than native-born children (*p* < 0.001).

The child-parent relationships of the study children are presented in Table 4. According to the results, girls more often reported supportive communication with parents than boys (80.2% versus 70.3%) (*p* < 0.001).

The associations between child-parent relationships and school engagement are presented in Table 5. High levels of school engagement were more prevalent in both boys (78.7%) and girls (85.1%) who had supportive communication with their parents than children with lower level of supportive communication (*p* < 0.001).

The results of the logistic regression analyses for the girls are presented in Table 6. These show positive associations between supportive communication and high levels of school engagement, in both the unadjusted (OR 2.47; 95% CI 2.34–2.60) and adjusted (OR 2.46; 95% CI 2.33–2.59) models. In addition, the results of the adjusted logistic regression analysis indicated that there were positive associations between a higher level of school engagement and being in the 4th grade (aged 9–10 years), living with both parents and coming from an immigrant background.

The results of logistic regression analyses for the boys are presented in Table 7. These show positive associations between supportive communication and high levels of school engagement in both the unadjusted (OR 2.10; 95% CI 2.01–2.19) and adjusted (OR 2.10; 95% CI 2.01–2.20) logistic regression models. In addition, according to the results, the adjusted logistic regression analysis showed positive associations between a high level of school engagement and being in the 4th grade (aged 9–10 years), living with both parents and coming from an immigrant background.

## 4. Discussion

This nationally representative, large-scale study provides new knowledge on the significant associations between supportive child-parent relationships and school engagement among children aged 9–11 years in Finland, including when controlling for the background factors.

This study indicated that it was more common for girls than boys to like school very much. Similar results have been found in previous national studies [4,5,6,7,20,61], an international study from 12 countries [19,30] and the WHO’s 2020 HBSC study [33]. There are several suggestions about why girls are more engaged at school than boys and one is that their high levels of school engagement are socially constructed. Secondly, the results can be affected by social desirability bias where the answers in self-reported questionnaire can be guided by social expectations more among girls than among boys [62]. Qualitative research is needed to generate more plausible explanations. It would also very beneficial to study whether there are differences between sub-groups of boys and girls when it comes school engagement.

High levels of school engagement were statistically significantly more prevalent among girls and boys in the 4th than 5th grades. These results are supported by previous studies indicating that school engagement declined as children moved to higher grades [20,27,61], and same result was found in a study that also examined girls and boys separately [7]. One explanation for this decline may be that 5th graders do not socially relate to their teachers as much as when they were in the 4th grade, as indicated by previous age-related research [63]. Furthermore, longitudinal data are needed to identify whether this difference represents real developmental trends across grades.

In this study, high levels of school engagement were more prevalent in girls and boys living with both parents, or in a shared residency arrangement, than children who lived with one parent. These results are supported by previous studies that indicated that lower levels of school engagement were more prevalent in children living in one-parent families than with both parents [35,38] or in shared residency [38,64]. Many factors have been suggested that could explain lower school engagement during shared residency and in one-parent families and these include stress and lower financial and social resources [65]. One explanation could be that the contact between the child and their parents is higher when they live with both parents or in shared residency, compared to when they live with one parent. That increased contact may lead to higher quality child-parent relationships [17,66]. However, the quality of child-parent relationships may only partially relate to the quantity of contacts, and to living arrangements. On the other hand, the presence of step-parents or parental partners in shared residency and one-parent families can increase children’s stress, which negatively affects school engagement [61]. More research is needed on the complex factors associated with the quality of child-parent relationships.

The results of this study show that high levels of school engagement were more prevalent in immigrant girls and boys, born in Finland and abroad, than in native-born children. This immigrant paradox has been reported by other studies [21,39]. In contrast, other studies showed that being from an immigrant background was associated with lower levels of school engagement [15,20,21,37,38]. How well immigrant children engaged with school reflects their short-term and long-term adaptation to society [37]. In one longitudinal study, school engagement decreased in immigrant students during the five-year follow up [67] and one explanation was that they disengaged from school over time to protect themselves from the negative psychological effects of school failure [21]. Supportive parenting has also have been positively associated with school engagement among immigrant children [3,17]. One suggestion is that the connection between parent and school engagement may be even more important for recently immigrated children than for native-born children [17]. More research is needed on the associations between immigrant child-parent relationships and school engagement.

In this study, girls were more likely to report supportive communication with their parents than boys. Supportive communication was associated with higher levels of school engagement, in both genders, than lower levels of supportive communication. The child-parent communication indicator included data on how often the children discussed their school day with their parents, how often children agreed with parents what time they would come home when they went out, how often the children’s parents talked with their friends when they meet them and how often the children’s parents supported and encouraged them. Previous studies have also indicated that discussing schoolwork with parents [7,13], family rules and boundaries [8,16] and supervision by parents [7,40,41,42,43,44] were factors that had a positive impact on the child’s school engagement.

School health services form the basis of health care for school-aged children and they provide a natural way of preventing social exclusion and supporting their well-being. In Finland they ensure broken continuity of health promotion after the services provided by child health clinics cease. They are also free of charge for students under the age of 18 [68]. Children are examined on an annual basis and more detailed health examinations are carried out when they are in 1st, 5th and 8th grades and are approximately seven, 11 and 14 years of age. Parents are invited to participate in these three comprehensive health examinations and one of the aims is to support the child-parent relationship and identify any problems [69].

According to the adjusted logistic regression model, supportive communication with parents was statistically significantly associated with high levels of school engagement among both girls and boys when we controlled for the background factors. High levels of school engagement were also more prevalent in girls than in boys and so was supportive communication between children and their parents. This raises question about whether girls have higher levels of school engagement because of higher quality child-parent relationships or because the school environment suits girls better than boys. According to a previous review, the fact that girls were better at adapting to school only partly explained their better academic achievements than boys [70]. More research is needed on this complex phenomenon in the future and the perspectives of the future research can be broadened by also using data gathered from the parents.

## 5. Limitations

This study had some limitations. The data provided by the Finnish School Health Promotion study were based on an 18-page questionnaire, which could be relatively long for children aged 9–11 years [56]. There is always the possibility of under and over reporting and some children can misunderstand questions in self-report questionnaires [59]. Secondly, the data were not collected from parents or the school environment which are known to be associated with school engagement [9]. The Cronbach’s alpha of the child-parent relationship sum variable was 0.59 which did not reach the satisfied alpha coefficient value of 0.70. However, the decisions made by scale affects the level of reliability that can be considered adequate [71]. The dichotomized child-parent communication indicator was used in the analysis instead of the sum variable. In addition, the school engagement was examined by one-item self-reported variable, which might limit the depth of the phenomena examined. However, since our dataset included questions similar to those used in other studies, such as the HBSC study, our results enable a comparison with their results. In addition, the one-item variable of school liking is used in several previous studies [63,72,73,74]. The strengths of this study were the nationally representative data, large sample size and relatively high response rate (82.0%).

## 6. Conclusions

This nationally representative large-scale study sought to examine the factors that were associated with school engagement among children aged 9–11 years in the 4th and 5th grades in Finnish primary schools. In addition, it examined child-parent relationships and their association with school engagement among both genders separately. High levels of school engagement were more prevalent among girls than boys and among both genders in the 4th than 5th grade.

Multivariate logistic regression indicated that supportive communication with parents was associated with higher levels of school engagement. In addition, being in the 4th grade, living with both parents and having an immigrant background were associated with higher levels of school engagement in both girls and boys.

These findings have some implications for parents and school community. The collaboration between home and school could include more provision of support for families to enable parents to get better involved with schooling of their children. One example of that could be mutual workshops for parents and educators in order to strengthen the pa-rental knowledge of school life. The results of this study also increased the scientific knowledge base on applied educational sciences; this may encourage the scholars in this field to utilize more secondary data on school well-being surveys in their research work.

More research is needed to identify the factors associated with supportive child-parent communication and identify the risk factors for lower levels of supportive communication. Supporting positive child-parent relationships would make it possible to improve school engagement and promote children’s well-being. This is important, because high levels of school engagement predict better academic achievement and higher life satisfaction in future life.

## Figures and Tables

**Table 1:** Background factors for girls and boys aged 9–11 years [49].

Variable	All	Girls	Boys	*p*-Value ^1^
	(*n* = 99,686)	(*n* = 49,650)	(*n* = 49,695)	
	% (*n*)	% (*n*)	% (*n*)	
School grade				0.181
4th grade (aged 9–10)	49.7 (49,360)	49.9 (24,722)	49.5 (24,522)	
5th grade (aged 10–11)	50.3 (49,992)	50.1 (24,830)	50.5 (25,051)	
Living arrangements				<0.001
Both parents at home	73.2 (70,377)	72.4 (35,020)	74.0 (35,177)	
Shared residency	20.5 (19,697)	20.9 (10,095)	20.1 (9551)	
One parent	4.2 (4067)	4.8 (2310)	3.7 (1743)	
Neither parent	2.1 (1995)	1.9 (936)	2.2 (1052)	
Immigrant status				0.006
Native born	86.3 (83,636)	86.1 (41,939)	86.6 (41,475)	
One parent immigrant (born Finland)	7.9 (7631)	7.9 (3851)	7.9 (3765)	
Both parents immigrants (born Finland)	2.9 (2819)	3.1 (1502)	2.7 (1309)	
Immigrant (born outside Finland)	2.9 (2801)	3.0 (1438)	2.8 (1347)	

^1^ chi-square test.

**Table 2:** School engagement reported by girls and boys aged 9–11 years [49].

Variable	All	Girls	Boys	*p*-Value ^1^
	(*n* = 99,686)	(*n* = 49,650)	(*n* = 49,695)	
	% (*n*)	% (*n*)	% (*n*)	
How much they liked school				<0.001
Very much	15.9 (15,773)	18.0 (8924)	13.8 (6810)	
Quite liked	62.0 (61,499)	63.9 (31,598)	60.2 (29,756)	
Little	18.9 (18,775)	16.2 (8001)	21.7 (10,707)	
Never	3.1 (3107)	1.9 (922)	4.4 (2160)	

^1^ chi-square test.

**Table 3:** Associations between the background factors and school engagement in girls and boys aged 9–11 years [49].

Variable	Girls with High Levels of School Engagement	*p*-Value ^1^	Boys with High Levels of School Engagement	*p*-Value ^1^
	% (*n*)		% (*n*)	
School grade		<0.001		<0.001
4th grade (aged 9–10)	84.0 (20,677)		75.0 (18,277)	
5th grade (aged 10–11)	79.9 (19,773)		73.0 (18,208)	
Living arrangements		<0.001		<0.001
Both parents at home	83.8 (29,223)		76.0 (26,595)	
Shared residency	77.9 (7837)		70.2 (6673)	
One parent	75.1 (1726)		68.1 (1182)	
Neither parent	75.6 (703)		66.7 (697)	
Immigrant status		<0.001		<0.001
Native born	81.8 (34,173)		73.8 (30,459)	
One parent immigrant (born Finland)	80.4 (3075)		74.4 (2784)	
Both parents immigrants (born Finland)	85.6 (1281)		81.7 (1061)	
Immigrant (born outside Finland)	88.3 (1264)		80.7 (1082)	

^1^ chi-square test.

**Table 4:** Child-parent relationships of girls and boys aged 9–11 years [49].

Variable	All	Girls	Boys	*p*-Value ^1^
	(*n* = 99,686)	(*n* = 49,650)	(*n* = 49,695)	
	% (*n*)	% (*n*)	% (*n*)	
Child-parent communicationindicator				<0.001
Supportive communication	75.2 (72,454)	80.2 (38,761)	70.3 (33,515)	
Lower level of supportive communication	24.8 (23,835)	19.8 (9589)	29.7 (14,174)	
Child discussed school day with parents				<0.001
Often	63.0 (61,835)	66.5 (32,751)	59.4 (28,936)	
Sometimes	34.3 (33,663)	31.3 (15,392)	37.3 (18,176)	
Never	2.8 (2727)	2.2 (1102)	3.3 (1612)	
Child agreed with parents what time they would come home after going out				<0.001
Often	60.4 (59,055)	66.5 (32,613)	54.3 (26,295)	
Sometimes	30.7 (29,985)	27.3 (13,395)	34.1 (16,502)	
Never	8.9 (8726)	6.2 (3052)	11.7 (5651)	
Parents talked with child’s friends when they met them				<0.001
Often	49.5 (48,288)	54.1 (26,494)	44.9 (21,670)	
Sometimes	40.8 (39,843)	38.4 (18,820)	43.3 (20,920)	
Never	9.6 (9409)	7.5 (3669)	11.8 (5714)	
Parents supported and encouraged				<0.001
Often	88.3 (85,906)	89.3 (43,622)	87.3 (42,075)	
Sometimes	10.4 (10,163)	9.7 (4743)	11.2 (5378)	
Never	1.2 (1208)	0.9 (460)	1.5 (745)	

^1^ chi-square test.

**Table 5:** Associations between child-parent relationships of children aged 9–11 and school engagement [49].

Variable	Girls with High Levels of School Engagement	*p*-Value ^1^	Boys with High Levels of School Engagement	*p*-Value ^1^
	% (*n*)		% (*n*)	
Child-parent communicationindicator		<0.001		<0.001
Supportive communication	85.1 (32,844)		78.7 (26,253)	
Lower level of supportive communication	69.8 (66,665)		63.8 (9001)	
Child discussed school day with parents		<0.001		<0.001
Often	85.5 (27,880)		78.5 (22,602)	
Sometimes	76.5 (11,729)		69.2 (12,514)	
Never	56.6 (621)		54.6 (875)	
Child agreed with parents what time they would come home after going out		<0.001		<0.001
Often	85.2 (27,678)		79.0 (20,655)	
Sometimes	77.0 (10,280)		70.4 (11,557)	
Never	70.1 (2128)		63.9 (3586)	
Parents talked with child’s friends when they met them		<0.001		<0.001
Often	85.1 (22,448)		78.7 (16,970)	
Sometimes	79.7 (14,934)		72.3 (15,056)	
Never	72.0 (2633)		64.6 (3668)	
Parents supported and encouraged		<0.001		<0.001
Often	84.2 (36,607)		76.8 (32,160)	
Sometimes	64.4 (3041)		58.2 (3110)	
Never	53.8 (245)		47.8 (355)	

^1^ chi-square test.

**Table 6:** ORs and 95% CIs for background factors and child-parent relationships of girls aged 9–11 years in relation to high levels of school engagement [49].

	Girls’ High Levels of School Engagement					
	OR ¹	Unadjusted 95% CI ^2^	*p*-Value	OR ¹	Adjusted 95% CI ^2^	*p*-Value
Child-parent communication indicator						
Supportive communication	2.47	2.34–2.60	<0.001	2.46	2.33–2.59	<0.001
Lower level of supportive communication	1			1		
School grade						
4th grade (aged 9–10)	1.32	1.26–1.38	<0.001	1.35	1.29–1.42	<0.001
5th grade (aged 10–11)	1			1		
Living arrangements						
Both parents at home	1.67	1.43–1.94	<0.001	1.54	1.31–1.60	<0.001
Shared residency	1.14	0.97–1.33	0.107	1.10	0.94–1.30	0.247
One parent	0.98	0.82–1.17	0.788	0.96	0.79–1.15	0.63
Neither parent	1			1		
Immigrant status						
Native born	1			1		
One parent immigrant (born Finland)	0.91	0.84–0.99	0.026	0.94	0.86–1.03	0.169
Both parents immigrants (born Finland)	1.33	1.14–1.53	<0.001	1.35	1.16–1.58	<0.001
Immigrant (born outside Finland)	1.67	1.42–1.97	<0.001	1.88	1.58–2.24	<0.001

OR ^1^, odds ratio; CI ^2^, confidence interval.

**Table 7:** ORs and 95% CIs for background factors and child-parent relationships of girls aged 9–11 years in relation to high level of school engagement [49].

	Boys’ High Levels of School Engagement					
	OR ¹	Unadjusted 95% CI ^2^	*p*-Value	OR¹	Adjusted 95% CI ^2^	*p*-Value
Child-parent communication indicator						
Supportive communication	2.10	2.01–2.19	<0.001	2.10	2.01–2.20	<0.001
Lower level of supportive communication	1			1		
Grade						
4th grade (aged 9–10)	1.11	1.07–1.15	<0.001	1.11	1.06–1.16	<0.001
5th grade (aged 10–11)	1			1		
Living arrangements						
Both parents at home	1.58	1.39–1.80	<0.001	1.43	1.24–1.65	<0.001
Shared residency	1.178	1.03–1.35	0.019	1.10	0.95–1.28	0.194
One parent	1.07	0.91–1.26	0.436	1.02	0.85–1.21	0.866
Neither parent	1			1		
Immigrant status						
Native born	1			1		
One parent immigrant (born Finland)	1.03	0.96–1.12	0.407	1.07	0.98–1.16	0.114
Both parents immigrants (born Finland)	1.59	1.38–1.83	<0.001	1.63	1.40–1.90	<0.001
Immigrant (born outside Finland)	1.48	1.29–1.70	<0.001	1.63	1.40–1.89	<0.001

OR ^1^, odds ratio; CI ^2^, confidence interval.

## References

[1] Jimerson S.R., Campos E., Greif J.L. (2003). Toward an understanding of definitions and measures of school engagement and related terms. Calif. Sch. Psychol..

[2] Fredricks J.A., Blumenfeld P.C., Paris A.H. (2004). School engagement: Potential of the concept, state of the evidence. Rev. Educ. Res..

[3] Wang M.-T., Willett J.B., Eccles J.S. (2011). The assessment of school engagement: Examining dimensionality and measurement invariance by gender and race/ethnicity. J. Sch. Psychol..

[4] Honma Y., Uchiyama I. (2014). Emotional engagement and school adjustment in late childhood: The relationship between school liking and school belonging in Japan. Psychol. Rep..

[5] Estell D.B., Perdue N.H. (2013). Social support and behavioral and affective school engagement: The effects of peers, parents, and teachers. Psychol. Sch..

[6] Raufelder D., Hoferichter F., Ringeisen T., Regner N., Jacke C. (2015). The perceived role of parental support and pressure in the interplay of test anxiety and school engagement among adolescents: Evidence for gender-specific relations. J. Child Fam. Stud..

[7] Wang M.-T., Eccles J.S. (2012). Social support matters: Longitudinal effects of social support on three dimensions of school engagement from middle to high school. Child Dev..

[8] Lanza H.I., Taylor R.D. (2010). Parenting in moderation: Family routine moderates the relation between school disengagement and delinquent behaviors among African American adolescents. Cult. Divers. Ethn. Minor. Psychol..

[9] Upadyaya K., Salmela-Aro K. (2013). Development of school engagement in association with academic success and well-being in varying social contexts: A review of empirical research. Eur. Psychol..

[10] Arora P.G., Wheeler L.A., Fisher S., Barnes J. (2017). A prospective examination of anxiety as a predictor of depressive symptoms among Asian American early adolescent youth: The role of parent, peer, and teacher support and school engagement. Cult. Divers. Ethn. Minor. Psychol..

[11] Portilla X.A., Ballard P.J., Adler N.E., Boyce W.T., Obradović J. (2014). An integrative view of school functioning: Transactions between self-regulation, school engagement, and teacher-child relationship quality. Child Dev..

[12] Kiiskilä K., Tuomaala S., Aunola K., Lerkkanen M.-K., Kiuru N. (2015). Associations between parenting styles and school related guid-ance and school engagement. Kasvatus.

[13] Wang M.-T., Sheikh-Khalil S. (2013). Does parental involvement matter for student achievement and mental health in high school?. Child Dev..

[14] Virtanen T.E., Lerkkanen M.-K., Poikkeus A.-M., Kuorelahti M. (2016). student engagement and school burnout in finnish lower-secondary schools: Latent profile analysis. Scand. J. Educ. Res..

[15] Hamilton M., Redmond G. (2019). Are young carers less engaged in school than non-carers? Evidence from a representative Australian study. Child Indic. Res..

[16] Chen B.-B., Wiium N., Dimitrova R., Chen N. (2017). The relationships between family, school and community support and boundaries and student engagement among chinese adolescents. Curr. Psychol..

[17] Garcia-Reid P., Peterson C.H., Reid R.J. (2013). parent and teacher support among Latino immigrant youth. Educ. Urban Soc..

[18] Jelas Z.M., Azman N., Zulnaidi H., Ahmad N.A. (2016). Learning support and academic achievement among Malaysian adolescents: The mediating role of student engagement. Learn. Environ. Res..

[19] Lam S.-F., Jimerson S., Shin H., Cefai C., Veiga F., Hatzichristou C., Polychroni F., Kikas E., Wong B.P.H., Stanculescu E. (2015). Cultural universality and specificity of student engagement in school: The results of an international study from 12 countries. Br. J. Educ. Psychol..

[20] Li Y., Lerner R.M. (2011). Trajectories of school engagement during adolescence: Implications for grades, depression, delinquency, and substance use. Dev. Psychol..

[21] Motti-Stefanidi F., Masten A., Asendorpf J.B. (2014). School engagement trajectories of immigrant youth. Int. J. Behav. Dev..

[22] Reyes M.R., Brackett M.A., Rivers S.E., White M., Salovey P. (2012). Classroom emotional climate, student engagement, and academic achievement. J. Educ. Psychol..

[23] Hakimzadeh R., Besharat M.-A., Khaleghinezhad S.A., Jahromi R.G. (2016). Peers’ perceived support, student engagement in academic activities and life satisfaction: A structural equation modeling approach. Sch. Psychol. Int..

[24] Finnish Institute for Health and Welfare Prevention of Exclusion. https://thl.fi/fi/web/lapset-nuoret-ja-perheet/hyvinvointi-ja-terveys/nuorten-syrjaytymisen-ehkaisy.

[25] Dunderfelt T. (2011). Lifespan Psychology.

[26] Bennett P. Clinical Psychology: Psychopathology through the Lifespan, 2015. https://web-a-ebscohost-com.libproxy.tuni.fi/ehost/detail/detail?vid=0&sid=14980212-bd81-4a4f-8c7b-23f3f46f0f57%40sdc-v-sessmgr02&bdata=JkF1dGhUeXBlPWNvb2tpZSxpcCx1aWQmc2l0ZT1laG9zdC1saXZlJnNjb3BlPXNpdGU%3d#AN=1099339&db=e000xww.

[27] Kokkonen P., Athanasopoulou C., Leino-Kilpi H., Sakellari E. (2021). Secondary school pupils’ mental wellbeing is associated with belonging to a perceived minority and experiencing discrimination. Children.

[28] Kelly A.B., O’Flaherty M., Toumbourou J.W., Homel R., Patton G.C., White A., Williams J. (2011). The influence of families on early adolescent school connectedness: Evidence that this association varies with adolescent involvement in peer drinking networks. J. Abnorm. Child Psychol..

[29] Ashiabi G.S. (2017). Perceptions of neighborhood characteristics and the perceived psychosocial and academic outcomes of U.S. children and adolescents. Int. J. Child Youth Fam. Stud..

[30] Jones G., LaFreniere K. (2012). Exploring the role of school engagement in predicting resilience among bahamian youth. J. Black Psychol..

[31] Lam S.-F., Jimerson S., Kikas E., Cefai C., Veiga F., Nelson B., Hatzichristou C., Polychroni F., Basnett J., Duck R. (2012). Do girls and boys perceive themselves as equally engaged in school? The results of an international study from 12 countries. J. Sch. Psychol..

[32] World Health Organization Health Policy for Children and Adolescents, No.5. https://www.euro.who.int/__data/assets/pdf_file/0005/53852/E91416.pdf.

[33] World Health Organization Liking School. https://gateway.euro.who.int/en/indicators/hbsc_42-liking-school/.

[34] OECD PISA Results: Sense of Belonging at School. https://www.oecd-ilibrary.org/sites/d69dc209-en/index.html?itemId=/content/component/d69dc209-en.

[35] Azagba S., Asbridge M., Langille D.B. (2014). Is religiosity positively associated with school connectedness: Evidence from high school students in Atlantic Canada?. J. Prim. Prev..

[36] Chiu M.M. (2007). Families, economies, cultures, and science achievement in 41 countries: Country-, school-, and student-level analyses. J. Fam. Psychol..

[37] Chiu M.M., Pong S.-L., Mori I., Chow B.W.-Y. (2012). Immigrant students’ emotional and cognitive engagement at school: A multilevel analysis of students in 41 countries. J. Youth Adolesc..

[38] Havermans N., Sodermans A.K., Matthijs K. (2017). Residential arrangements and children’s school engagement. Youth Soc..

[39] Motti-Stefanidi F., Masten A. (2013). School success and school engagement of immigrant children and adolescents. Eur. Psychol..

[40] Blondal K.S., Adalbjarnardóttir S. (2014). Parenting in relation to school dropout through student engagement: A longitudinal study. J. Marriage Fam..

[41] Chilenski S.M., Ridenour T., Bequette A.W., Caldwell A.W.B.L. (2015). Pathways of influence: How parental behaviors and free time experiences are associated with African American early adolescent development and academic achievement. J. Negro Educ..

[42] Dotterer A.M., Wehrspann E. (2016). Parental knowledge: Examining reporter discrepancies and links to school engagement among middle school studies. J. Youth Adolesc..

[43] Hill N.E., Wang M.-T. (2015). From middle school to college: Developing aspirations, promoting engagement, and indirect pathways from parenting to post high school enrollment. Dev. Psychol..

[44] Voisin D.R., Harty J., Kim D.H., Elsaesser C., Takahashi L. (2017). Assessing the relationship between parental influences and wellbeing among low income African American adolescents in Chicago. Child Youth Care Forum.

[45] Jaggers J.W., Bolland A.C., Tomek S., Bolland K.A., Hooper L.M., Ii W.T.C., Bolland J.M. (2018). The longitudinal impact of distal, non-familial relationships on parental monitoring: Implications for delinquent behavior. Youth Soc..

[46] Bartle-Haring S., Younkin F.L., Day R. (2012). Family distance regulation and school engagement in middle-school-aged children. Fam. Relat..

[47] Ministry of Education and Culture (2021). Finnish Education System. https://minedu.fi/en/education-system.

[48] Finnish Institute for Health and Welfare Response Rates. https://thl.fi/fi/tutkimus-ja-kehittaminen/tutkimukset-ja-hankkeet/kouluterveyskysely/vastaajamaara#kunnat.

[49] Finnish Institute for Health and Welfare Well-Being of Children and Young People: School Health Promotion Study. http://urn.fi/URN:NBN:fi-fe2019091528281.

[50] Finnish Institute for Health and Welfare School Health Promotion Study. https://thl.fi/en/web/thlfi-en/research-and-development/research-and-projects/school-health-promotion-study.

[51] Finnish Institute for Health and Welfare Instructions for Institution. https://thl.fi/fi/tutkimus-ja-kehittaminen/tutkimukset-ja-hankkeet/kouluterveyskysely/kouluterveyskyselyn-toteuttaminen/ohjeet-oppilaitoksille.

[52] InfoFinland.fi Comprehensive Education. https://www.infofinland.fi/en/living-in-finland/education/child-education/comprehensive-education.

[53] Finnish National Board on Research Integrity TENK Responsible Conduct of Research and Procedures for Handling Allegations of Misconduct in Finland. Guidelines of the Finnish Advisory Board on Research Integrity 2012..

[54] Finnish National Board on Research Integrity TENK The Ethical Principles of Research with Human Participants and Ethical Review in the Human Sciences in Finland. https://tenk.fi/sites/default/files/2021-01/Ethical_review_in_human_sciences_2020.pdf.

[55] Finnish Institute for Health and Welfare Execution of the School Health Promotion Study. https://thl.fi/fi/tutkimus-ja-kehittaminen/tutkimukset-ja-hankkeet/kouluterveyskysely/kouluterveyskyselyn-toteuttaminen.

[56] Finnish Institute for Health and Welfare School Health Promotion Study 2017. https://sampo.thl.fi/pivot/prod/fi/ktk/ktk4/summary_perustulokset2?alue_0=87869&mittarit_0=200010&mittarit_1=199261&mittarit_2=200163&vuosi_0=v2017#.

[57] Finnish Institute for Health and Welfare School Health Promotion Study 2019, 4th and 5th Grade Students. https://thl.fi/documents/605877/4438922/Perusopetuksen_4-5_lk_lomake_FI_mallikappale.pdf/93a1d839-4e65-4e7f-a81a-d2d6840ab034.

[58] Nummenmaa L., Holopainen M., Pulkkinen P. (2016). Basics of Quantitative Research.

[59] Valli R. Initiation into Quantitative Research. https://www.ellibslibrary.com/reader/9789524516761.

[60] Hosmer D., Lemeshow S., Sturdivant R. (2013). Applied Logistic Regression. https://ebookcentral.proquest.com/lib/tampere/detail.action?docID=1138225&pq-origsite=primo.

[61] Havermans N., Vanassche S., Matthijs K. (2017). Children’s post-divorce living arrangements and school engagement: Financial resources, parent–child relationship, selectivity and stress. J. Child Fam. Stud..

[62] Lauglo J., Liu F. (2019). The reverse gender gap in adolescents’ expectation of higher education: Analysis of 50 education systems. Comp. Educ. Rev..

[63] Gest S.D., Welsh J.A., Domitrovich C.E. (2005). Behavioral predictors of changes in social relatedness and liking school in elementary school. J. Sch. Psychol..

[64] Gore J.S., Thomas J., Jones S., Mahoney L., Dukes K., Treadway J. (2015). Social factors that predict fear of academic success. Educ. Rev..

[65] Havermans N., Botterman S., Matthijs K. (2014). Family resources as mediators in the relation between divorce and children’s school engagement. Soc. Sci. J..

[66] Bauserman R. (2002). Child adjustment in joint-custody versus sole-custody arrangements: A meta-analytic review. J. Fam. Psychol..

[67] Suárez-Orozco C., Rhodes J., Milburn M. (2009). Unraveling the immigrant paradox. Youth Soc..

[68] Ministry of Social Affairs and Health Primary Health Care. https://stm.fi/en/primary-health-care.

[69] Finnish Institute for Health and Welfare Content and Purpose of Heath Examinations. https://thl.fi/fi/web/lapset-nuoret-ja-perheet/sote-palvelut/opiskeluhuolto/kouluterveydenhuolto/terveystarkastukset/terveystarkastusten-sisalto-ja-tarkoitus.

[70] Spinath B., Eckert C., Steinmayr R. (2014). Gender differences in school success: What are the roles of students’ intelligence, personality and motivation?. Educ. Res..

[71] Cortina J.M. (1993). What is coefficient alpha? An examination of theory and applications. J. Appl. Psychol..

[72] Jerdén L., Burell G., Stenlund H., Weinehall L., Bergström E. (2011). Gender differences and predictors of self-rated health development among swedish adolescents. J. Adolesc. Health.

[73] Løhre A., Moksnes U.K., Lillefjell M. (2014). Gender differences in predictors of school wellbeing?. Health Educ. J..

[74] Rönkä A.R., Sunnari V., Rautio A., Koiranen M., Taanila A. (2017). Associations between school liking, loneliness and social relations among adolescents: Northern Finland Birth Cohort 1986 study. Int. J. Adolesc. Youth.

[75] Finnish Institute for Health and Welfare (2020). Kouluterveyskysely. www.thl.fi/kouluterveyskysely.

